# Genetic Variation and the Role of Multigene Panel Testing for Hereditary Breast Cancer: A Single-Institution Experience

**DOI:** 10.7759/cureus.14637

**Published:** 2021-04-22

**Authors:** Kit Lu, Meagan Smith, Tejaswi Kanderi, Julia Verbiar, Jennifer Laspe,, Latesha Bair, Lisa Torp

**Affiliations:** 1 Medical Oncology, University of Pittsburgh Medical Center Hillman Cancer Center, Harrisburg, USA; 2 Genetics, University of Pittsburgh Medical Center Hillman Cancer Center, Harrisburg, USA; 3 Internal Medicine, University of Pittsburgh Medical Center Harrisburg, Harrisburg, USA; 4 Medicine, University of Pittsburgh Medical Center Hillman Cancer Center, Harrisburg, USA; 5 Breast Care Center, University of Pittsburgh Medical Center Hillman Cancer Center, Harrisburg, USA

**Keywords:** genetic variation, breast cancer, extended panel, retrospective study

## Abstract

Background

Breast cancer is the second leading cause of cancer death in women. There are multiple pathogenic mutations in addition to BRCA1/2 that are implicated in causing hereditary breast cancer.

Methods and results

We conducted a retrospective analysis of 1568 patients with breast cancer diagnosed between January 1, 2015, and December 31, 2018. The age range is 23-87. Among the study population, 26% had genetic testing and 8% of those were found to carry a pathogenic variant, as designated in NCCN (National Comprehensive Cancer Network) Guidelines. Of that 8%, 3.4% were BRCA1 and BRCA2 mutations, and the rest were other prevalent pathogenic variants.

Discussion

Expanded panel testing has the potential to increase the detection rate of pathogenic variants compared to testing for BRCA1/2 alone. Diagnostic accuracy of genetic causes of breast cancer has a significant clinical impact on patients and their families in terms of targeted treatment and prevention strategies. There is a strong need for further understanding of genetic patterns and variations in hereditary breast cancer. Awareness of the possibility of moderate to low penetrance genes and variants of uncertain significance (VUS) is important to assist with appropriate genetic counseling. We believe that physicians should consider re-testing with an expanded panel if patients previously had BRCA1 and BRCA2 testings only with a negative result as it may identify additional mutations.

## Introduction

Breast cancer is the second leading cause of cancer death in women. Although most breast cancers are sporadic, an estimated 5-10% are due to hereditary causes [1,2]. There are multiple pathogenic mutations in addition to BRCA1/2 that are implicated in causing hereditary breast cancer [3].

Studies have shown that 45% of breast cancer patients with pathogenic germline variants are being missed if testing is performed according to prior NCCN (National Comprehensive Cancer Network) guidelines with restricted panels. NCCN has modified the criteria for customized panel testing with the use of the multi-gene panel, useful for not only breast cancer but also other cancers and syndromes associated with cancers. Genes such as CDH1, MSH2, MLH1, MSH6, PMS2, PTEN, STK11, and TP53 have corresponding current management suggestions in the NCCN guidelines. However, other moderate penetrance genes like BARD1, RAD50, ATM, BRIP1, CHEK2, NBN, PALB2, RAD51C do not have specific treatment guidelines which contribute to complexity requiring the extensive need for genetic counseling both pre- and post-test [4]. Recent studies have shown an increase in the diagnosis of hereditary breast cancer with the use of multigene panels compared to the restricted gene panel, and guidelines now recommend genetic testing for all patients with breast cancer as well as multigene testing for those with negative BRCA1/2 testing [5].

Under these guidelines, we report a single institutional experience highlighting the use and outcomes of multigene panel testing.

## Materials and methods

A retrospective analysis of germline multigene testing was performed including 1568 males and females who were diagnosed with breast cancer and/or being treated at our Cancer Center between January 1, 2015, and December 31, 2018. Variables included the following: age at diagnosis, personal medical history, significant family history, and genetic testing. Data were obtained via electronic medical record review. No patient identifiers were included. The study was approved by the UPMC Pinnacle Institutional Review Board.

## Results

**Figure 1:**
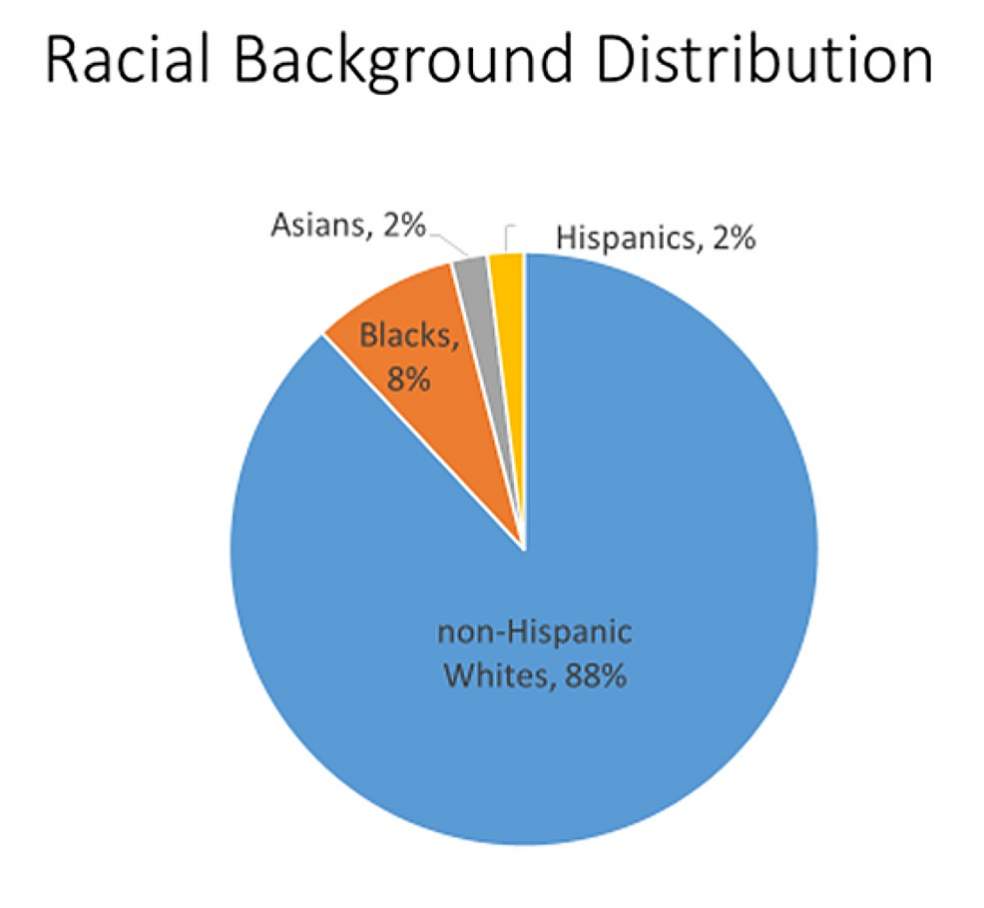
Pie chart representing the racial distribution of the study population

**Figure 2:**
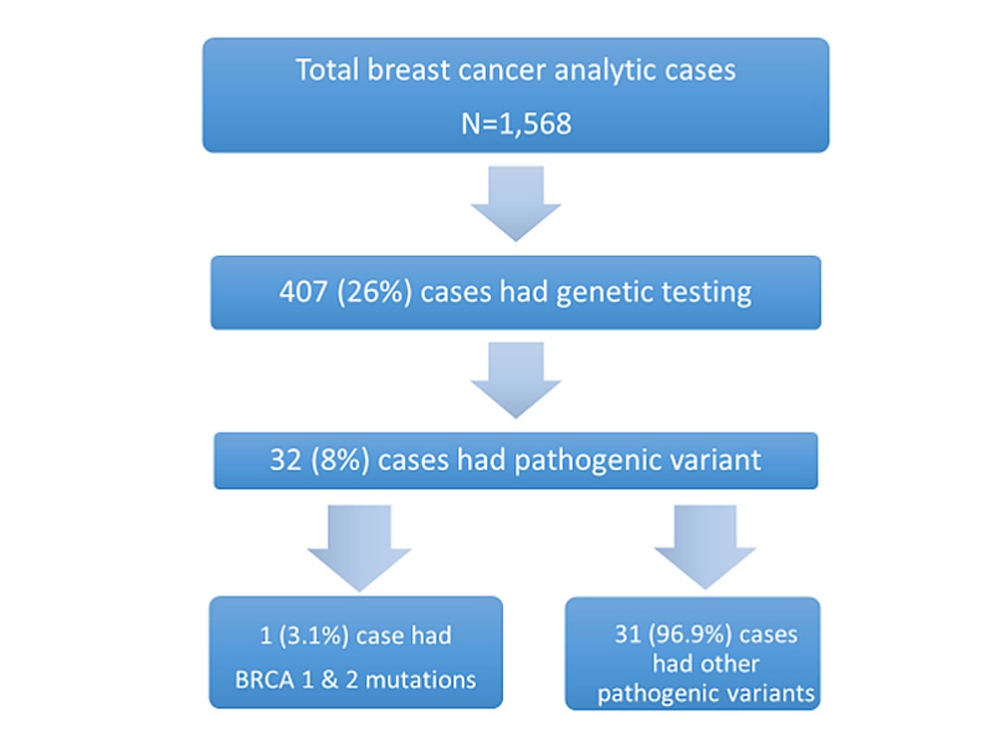
Flow chart depicting the results of retrospective analysis

The study patients ranged in age from 23 to 87 years with a median age of 52 years. Among the patient population, 88% are non-Hispanic whites, 8% are blacks, 2% percent are Asians, 2% are Hispanics, and less than 1% are male as represented in Figure 1. Of our total analyzed breast cancer cases, only 26% of patients had genetic testing. Among them, 8% of the patients were found to carry a pathogenic variant that NCCN Guidelines have designated as having enough evidence to confer an increased risk of breast cancer. Of that, only 3.4% were BRCA1 and BRCA2 mutations as represented in Figure 2. The other prevalent pathogenic variants are as follows: CHEK 2 (2.2%), PALB2 (1%), TP 53 (1%), PMS2 (1%), MYTYH (0.74%), FANCC (0.25 %), ATM (0.25 %), MSH2 (0.25 %), MSH6 (0.25 %), NBN (0.25 %), and RAD51c (0.25 %). Among the study population, 15 patients had initial testing only for BRCA1/2. Updated, expanded testing identified an additional two patients with a pathogenic variant that was unidentified on initial testing.

## Discussion

Although BRCA1 and BRCA2 are the most mutated genes, additional genes associated with hereditary breast cancer are emerging [6]. Many prospective and retrospective studies have been on the utility of expanded gene testing with the idea of accurately identifying pathogenic, likely pathogenic, or variants of uncertain significance (VUS) and influencing clinical management decisions.

Research has identified mutations in moderate penetrance genes like CHEK2, PALB2, ATM, BIRP1, RAD51C, XRCC2, BARD1 in addition to high penetrance genes such as BRCA1/2, P53, PTEN, STK11, CDH1 attributing to the significant familial risk of breast cancer [7]. Knowing the genetic defect helps to guide targeted therapies with accurate and beneficial results, however, recommendations like extreme prevention strategies like prophylactic surgeries need extensive data [8].

A prospective study performed using a 44 gene panel testing including patients with breast cancer, colorectal cancer, and other early-onset cancers who have tested negative for pathogenic genes including BACA1/2 showed that 5% of the study population carried pathogenic or highly pathogenic variants of moderate to high penetrance genes like BRCA2, MSH6, CHECK2, ATM, and MUTYH and 87 VUS. Seventy-seven percent of pathogenic and likely pathogenic variants were identified in breast cancer individuals highlighting the importance of multigene testing for identifying variants and VUS in familial breast cancer patients [9]. A retrospective study among patients with hereditary breast and ovarian cancer with no pathogenic BRCA mutations on initial testing, identified pathogenic/likely pathogenic mutations in genes like PALB2, BRIP1, BARD1, and RAD50 in addition to VUS in TP53, CHEK2, and CDH1 genes [10]. The inclusion of PALB2 among multigene panels is supported by studies revealing pathogenic/likely pathogenic mutations in the gene [4].

Another study with 89% of the population with breast cancer used next-generation sequencing analysis with a 94-gene panel and showed a total of 81 pathogenic/likely pathogenic mutations in 29% of patients including mutations in BRCA1, BRCA2. Genes were present in patients who were negative for BRCA1/2 on initial testing [11].

A study conducted using the Clinical Genome Resource clinical validity framework to assess the strength of evidence between selected genes in panel tests and breast or ovarian cancer did not reveal a convincing gene-disease relationship thus raising the importance of tailoring appropriate gene panel testing for eligible patients rather than applying it to a wide group of patients to avoid unnecessary testing including detection of VUS [12].

Studies also showed protein-truncating variants and missense variants of known genes predicting risk for breast cancer. Association between protein-truncating variants and breast cancer risk among genes like BRCA1/2, TP53, PTEN, CDH1, STK11, NF1, PALB2, ATM, CHEK2, and NBN was established [3]. While mutations in BRCA1/2 predict high risk, PALB2, PTEN, CHEK2, ATM, and NF1 predict moderate risk [13].

The difference in frequency of pathogenic/likely pathogenic and VUS was observed among different racial and ethnic groups in a retrospective cohort study of patients diagnosed with breast cancer at age <50 years [14]. Research stratifying different groups of the population is an area to be explored to formulate specific guidelines.

Genetic counseling for most of the newly identified genes is quite complicated [8,15]. Studies also have demonstrated that different labs identify different variants; some labs might identify a particular gene as a variant of uncertain significance, others might identify the same gene as pathogenic due to lack of sufficient evidence to prove either classification [16]. Extensive studies with large cohorts utilizing expanded genetic testing can provide clear and definitive guidelines and avoid discrepancies among labs.

With genetic testing becoming more affordable, there is always a chance of identifying VUS with no definite clinical implication causing anxiety and burden among patients and their families. Therefore, careful weighing of detailed risk versus benefits of testing is essential [3,17].

There is an obvious need for a large well-designed study involving a diverse population to identify other genes that play a role in breast cancer and to accurately assess the risk of hereditary breast cancer. Also collecting data from available panel testing will provide important information [13].

Limitations

Our study is the representation of the patient population treated in our institution during the specified timeline and does not represent the general population. Also, a larger cohort of the population is needed to further increase the power of the study.

## Conclusions

Multi-gene testing and expanded panel testing of all breast cancer patients could increase the detection rate of pathogenic variants compared to testing for BRCA1/2 alone and may have a potential clinical impact for patients and their families in terms of targeted treatment and prevention strategies. Further understanding of genetic patterns and variation in multi-gene testing is needed. Increasing public awareness and decreasing the cost of genetic testing is also important to improve the rates of multi-gene testing. As the costs of sequencing decrease, the concept of whole-genome sequencing can become widespread, making multi-gene panels affordable both as an initial test and a re-test among patients with incomplete testing.

Through our study, we demonstrate evidence in support of multi-gene panel testing for hereditary breast cancer. Physicians should be made aware of the possibility of moderate to low penetrance genes and VUS to assist with genetic counseling appropriately. Finally, physicians should consider retesting with the expanded panel if patients previously had BRCA1 and BRCA2 testings with a negative result as we have identified additional mutations upon retesting.
